# PAPP-A Protein Diagnostic and Prognostic Significance in Acute Coronary Syndromes Without Persistent ST-T-Segment Elevation

**DOI:** 10.3390/jcm15041455

**Published:** 2026-02-12

**Authors:** Monika Różycka-Kosmalska, Rafał Frankowski, Mikołaj Grabarczyk, Kasper Sipowicz, Anna Pękala-Wojciechowska, Tadeusz Pietras, Grzegorz Opielak, Marcin Kosmalski

**Affiliations:** 1 Department of Clinical Electrocardiology, Medical University of Lodz, 92-213 Lodz, Poland; monika.rozycka-kosmalska@umed.lodz.pl; 2 Department of Clinical Pharmacology, Medical University of Lodz, 90-153 Lodz, Poland; rafal.frankowski@student.umed.lodz.pl (R.F.); mikolaj.grabarczyk@stud.umed.lodz.pl (M.G.); anna.pekala-wojciechowska@umed.lodz.pl (A.P.-W.); tadeusz.pietras@umed.lodz.pl (T.P.); 3 Department of Interdisciplinary Research in the Area of Social Inclusion, The Maria Grzegorzewska University, 02-353 Warsaw, Poland; ksipowicz@aps.edu.pl; 4 Department of Human Physiology of the Chair of Preclinical Sciences, Medical University of Lublin, 20-080 Lublin, Poland; grzegorz.opielak@umlub.pl

**Keywords:** acute coronary syndrome, PAPP-A, ACS, cardiology, cardiovascular disease

## Abstract

**Background:** There are ongoing attempts to find a reliable, highly sensitive and specific early indicator of myocardial ischemia. Recently, a potential new function for the “non-pregnancy”-related pregnancy-associated plasma protein-A (PAPP-A) protein has been reported in many papers, including that the protein could be used in diagnosing heart conditions. Hence, our study aimed to determine the diagnostic and prognostic significance of PAPP-A protein in individuals diagnosed with non-ST-elevation acute coronary syndromes (NSTE-ACSs). **Methods:** The study comprised 100 consecutive patients (68 males and 32 females), aged from 42 to 83 years (mean age: 64.2 years). We assessed PAPP-A protein levels, anthropometric measurements, basic laboratory tests, ECG recordings, and coronary angiography for each patient. The participants were subsequently divided into two groups: non-ST-elevation myocardial infarction (NSTEMI, *n* = 74) or unstable angina (UA, *n* = 25). **Results:** The levels of PAPP-A protein in patients with NSTEMI were slightly higher than those in patients with UA, but the difference was not statistically significant (7.93 ± 6.35 mIU/L vs. 6.52 ± 5.45 mIU/L, *p* = 0.253). Higher PAPP-A protein levels (≥5.83 mIU/L) were associated with a numerically higher, but not statistically significant, risk of NSTEMI (OR = 1.37; 95% CI: 0.56–3.36). After 12 months, there was a significant correlation between the amount of labelled PAPP-A protein and the likelihood of experiencing acute myocardial infarction, cardiovascular death, and the necessity for unplanned coronary angiography. **Conclusions:** The diagnostic utility of PAPP-A protein in NSTE-ACS is limited, both in the NSTEMI and UA patient groups. However, its measurement can be used to estimate the annual risk for these groups of patients.

## 1. Introduction

Cardiovascular diseases (CVDs) are the primary cause of mortality in the human population. Their high prevalence is driven by widespread exposure to major risk factors such as smoking, high-cholesterol diet, sedentary lifestyle and environmental pollution [1,2]. The global burden of CVDs is projected to rise further in the upcoming decades, reaching an estimated 1.14 billion cases in 2050 [2,3]. Among CVDs, acute coronary syndromes (ACSs) are especially dangerous. In these emergency states, coronary blood flow is abruptly reduced due to vasoconstriction, embolic obstruction, disruption of an atherosclerotic plaque and subsequent thrombosis. Without timely intervention, this may result in cardiomyocyte necrosis and death, often due to critical heart rhythm (HR) abnormalities and heart failure (HF) [4,5]. For this reason, improving diagnostic tools and algorithms remains essential to enhance detection, differentiation and optimal personalized treatment regimens for patients at elevated risk of ACS complications and recurrent ischemia [3,4]. A 12-lead electrocardiogram (ECG) is the basis for classifying patients with suspected ACS symptoms (most commonly chest pain) into ST-segment elevation ACS (STE-ACS) and non-ST-elevation ACS (NSTE-ACS) [4,6]. A second axis of risk stratification involves measurement of biomarkers of myocardial injury and necrosis, primarily cardiac troponins (cTn) [5,6,7]. In recent years, pregnancy-associated plasma protein A (PAPP-A) has gained attention as a factor involved in CVD pathology and a potential diagnostic marker for distinguishing stable ischemic heart disease (IHD) from ACSs [8]. PAPP-A is a high-molecular-weight glycoprotein (~200 kDa) with zinc-binding metalloproteinase activity, and its main sources in the cardiovascular system include endothelium and arterial smooth muscle cells [9,10,11,12]. Notably, the form detected in ACSs differs from the pregnancy-associated complex. In ACSs it is present primarily as a homodimer composed of two subunits not bound to the proform of eosinophil major basic protein (proMBP) [13,14]. This difference is likely driven by oxidative conditions within unstable atherosclerotic plaques, which prevent formation of the covalent proMBP–PAPP-A complex [15,16]. This circulating PAPP-A form has been linked to several pathophysiological processes including disturbance of carbohydrate metabolism and coronary calcification [17]. Immunohistochemical and molecular analyses have also demonstrated elevated PAPP-A expression within unstable and ruptured carotid atherosclerotic plaques where it potentially contributes to plaque destabilization through extracellular matrix degradation and direct or indirect (insulin-like growth factor 1 (IGF-1)-mediated) promotion of inflammatory pathways [8,11,18,19,20,21,22,23]. Alternative interpretations propose that PAPP-A may primarily reflect intense cell proliferation and repair in unstable plaques rather than actively driving their disruption [24,25]. Regardless of the mechanism, these associations make PAPP-A a promising target in ACS cohorts, as plaque instability is a key driver of coronary events. Plasma PAPP-A concentrations have been reported to increase within 2–30 h after onset of chest pain of coronary origin [26]. Available data indicate that PAPP-A elevation may occur independently of cTn and creatine kinase MB (CK-MB), suggesting potential value when necrosis markers are nondiagnostic or ECG findings are inconclusive [27,28]. Moreover, multiple studies report associations between upregulated plasma PAPP-A and increased risk of mortality, HF, adverse cardiovascular events, and subsequent coronary interventions after ACS [11,27,28,29,30,31,32]. The purpose of this study was to determine the diagnostic and prognostic value of PAPP-A protein in patients with NSTE-ACS and to evaluate whether measuring PAPP-A can support risk stratification in this population. The specific objectives were as follows:

- To assess whether PAPP-A protein determination has diagnostic significance in identifying acute myocardial infarction (AMI).
- To compare the sensitivity, specificity and predictive value of PAPP-A protein with the classic marker of myocardial necrosis—cardiac troponin T (cTnT).
- To assess whether PAPP-A is a significant predictor of adverse cardiovascular events, including cardiovascular death (CD) and AMI, during follow-up at 1, 3, 6 and 12 months.
- To assess whether PAPP-A has prognostic value for the occurrence of restenosis.

## 2. Materials and Methods

### 2.1. Study Population

The Bioethics Committee of the Medical University of Lodz approved the study protocol (RNN/2/08/KE). The study included 100 patients with an initial diagnosis of NSTE-ACS treated in the Department of Cardiology, Medical University of Lodz, from 2008 to 2009. The inclusion criteria for the study were as follows: clinical diagnosis of ACS within 12 h from the onset of pain, electrocardiographic features of NSTE-ASC, including transient ST-segment elevation, new horizontal or downward sloping ST-segment depression ≥ 0.5 mV in two adjacent leads, T-wave inversion ≥ 0.1 mV or “pseudo-normalization” of T-waves, absence of ischemic changes on resting ECG, and informed consent to participate in the study signed by the patient. The exclusion criteria included a diagnosis of STE-ACS, NSTE-ACS diagnosed more than 12 h after symptom onset, renal failure (creatinine > 1.6 mg/dL), recent illness or severe skeletal muscle injury within the days preceding admission to hospital, acute or chronic infectious diseases, and pregnancy. Treatment with unfractionated or low-molecular-weight heparin was also considered as an exclusion condition because heparin is known to cause the PAPP-A protein to rapidly enter the circulation, resulting in its falsely elevated levels in the blood [33]. Patients enrolled in the study were interviewed, and their medical history was obtained to characterize the demographic and clinical features of the studied population (age, sex, smoking status, history of diabetes, hypertension, dyslipidaemia and familial occurrence of atherosclerotic diseases). During physical examination, weight and height were measured and used to calculate body mass index (BMI). Data from coronary angiography (location and number of hemodynamically significant stenoses due to atherosclerotic plaques) and information on treatment strategy (conservative management, percutaneous coronary intervention (PCI), or coronary artery bypass grafting (CABG)) were also collected. All patients enrolled in the study received standardized pharmacological therapy during the initial hospitalization and continued the same treatment after discharge throughout the follow-up period. Antiplatelet therapy consisted of acetylsalicylic acid (75 mg once daily) and clopidogrel (75 mg once daily). In addition, all patients were treated with a beta-adrenergic blocker (metoprolol succinate, 25–100 mg once daily), a statin (simvastatin, 40 mg once daily), an angiotensin-converting enzyme inhibitor (ramipril, 2.5–5 mg once daily), and trimetazidine (35 mg twice daily). This pharmacological regimen was maintained uniformly in all participants, and no patient discontinued or modified the prescribed therapy during the observation period. In each case of PCI, a bare-metal stent was implanted. Stent dimensions were selected at the discretion of the interventional cardiologist based on angiographic assessment of the target lesion. The implanted stents had diameters ranging from 3.0 to 3.5 mm and lengths ranging from 15 to 34 mm. No drug-eluting stents were used in the study population.

### 2.2. Biochemical Analysis of Blood Samples

Blood samples were collected on admission to hospital, within 12 h of the onset of pain, to assess routine biochemical parameters (glycemia, lipid profile, creatinine, uric acid, C-reactive protein (CRP), fibrinogen, and complete blood count) and cTnT, a marker of cardiomyocyte death. Measurements were performed using the COBAS INTEGRA 400 plus analyzer (Roche Diagnostic GmbH, Mannheim, Germany). A positive result of cTnT measurement was considered according to manufacturer-defined cut-offs (0.014 ng/mL). An additional blood sample of 5 mL was collected for quantification of PAPP-A. PAPP-A was measured using the ultrasensitive (US) Enzyme-Linked Immunosorbent Assay (ELISA) reagent (DRG Instruments GmbH, Marburg, Germany) based on the classical ELISA principle. This method detects specific proteins in biological material using mono- or polyclonal antibodies conjugated to the appropriate enzyme [34,35,36]. The assay employed the “sandwich” ELISA format.

### 2.3. Echocardiographic Examination

All patients underwent echocardiographic examination of ejection fraction (EF) performed with a Sonos 5500 or Sonos 2000 device (Hewlett-Packard Company, Palo Alto, CA, USA) with standard multiplanar 3.5 Hz transducers.

### 2.4. Clinical Follow-Up Period

Participants were followed clinically for 12 months. They were interviewed four times (after 1, 3, 6, and 12 months) regarding their current health status. Follow-up information was obtained by telephone, during outpatient visits when applicable, and through in-person home visits. Particular attention was paid to restenosis (significant luminal narrowing ≥50% at the site of a previous PCI), the need for unplanned coronary angiography (UCA), and adverse events including recurrent AMI (diagnosed according to universal definitions of myocardial injury and myocardial ischemia) or CD—death due to cardiovascular causes such as AMI, stroke, HF, or sudden cardiac arrest [3,37,38]. All assessed endpoints were adjudicated in a blinded manner. Patients with clinical suspicion of restenosis underwent angiographic assessment to confirm the occurrence of this adverse event.

### 2.5. Statistical Analysis

Quantitative variables are presented using descriptive statistics, including the arithmetic mean and standard deviation (SD), minimum and maximum values, and positional measures (median and the 25th and 75th percentiles (Q1 and Q3)). Qualitative (nominal) variables are reported as counts (*n*) and percentages (%). The choice of statistical methods for quantitative variables depended primarily on their distribution; therefore, normality was assessed using the Shapiro–Wilk test. If the distribution of a variable was non-normal in at least one group, between-group comparisons were performed using the non-parametric Mann–Whitney U test. If there was no basis to reject the assumption of normality, Student’s t-test was used to compare groups. The chi-square test of independence was applied to compare groups with respect to qualitative variables. The diagnostic and prognostic usefulness of PAPP-A was evaluated using receiver operating characteristic (ROC) curve analysis to assess its ability to differentiate between NSTEMI and UA and to predict adverse events at 12-month follow-up. A higher area under the ROC curve (AUC) indicates better discriminatory performance. Based on the ROC curve, optimal cut-off values for PAPP-A were determined to predict AMI and adverse events. We also calculated standard indices of diagnostic performance, including sensitivity, specificity, false-positive (FP) rate, false-negative (FN) rate, positive predictive value (PPV), and negative predictive value (NPV). The association between a positive test result and the occurrence of cardiovascular events was additionally expressed using the odds ratio (OR). Correlations between quantitative variables were assessed using Spearman’s rank correlation coefficient (R). A significance level of *p* = 0.05 was adopted for all tests. Statistical analyses were performed using STATISTICA PL 7.1 software (StatSoft, Tulsa, OK, USA).

## 3. Results

The study included 100 patients aged 42 to 83 years with a diagnosis of ACS [68 men (mean age 63.4 ± 10.3 years) and 32 women (mean age 66 ± 11.03 years); *p* = 0.252]. Ultimately 74 participants were diagnosed with NSTEMI and the remaining 26 with UA. Subsequent analyses were performed after stratifying patients into the NSTEMI and UA groups. The NSTEMI and UA groups did not differ significantly with respect to demographic characteristics, EF or the results of routine biochemical tests obtained on admission [Table 1]. Selective coronary angiography was performed via radial access in 83 patients and via femoral access in 17 patients. Five patients subsequently underwent CABG. Hemodynamically significant coronary stenosis was significantly more frequent in NSTEMI than in UA patients. The groups did not differ in the distribution of one-, two-, or three-vessel disease. In contrast, nearly one-quarter of UA patients had only marginal irregularities [Table 1]. Based on angiographic findings, patients were qualified for conservative treatment (17%) or invasive/surgical management (83%). Patients with UA were significantly more often assigned to conservative treatment (30.8% vs. 12.2%, *p* = 0.029).

### 3.1. The Role of PAPP-A Protein in Predicting Myocardial Infarction

**Figure 1:**
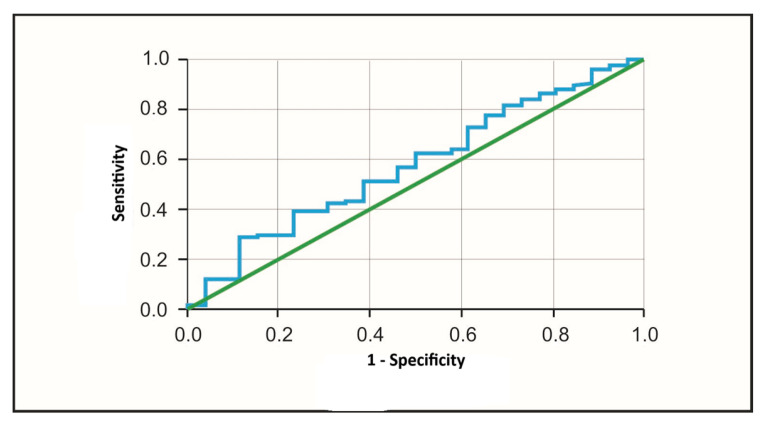
ROC curve—tested variable: PAPP-A protein concentration in differentiating patients with NSTEMI and UA.

We observed slightly higher mean and median PAPP-A protein concentrations in patients with NSTEMI compared to those with UA, but these differences were not statistically significant [Table 2]. The ROC curve, a graphical presentation of changes in the sensitivity and specificity of a given factor, described the usefulness of PAPP-A protein concentration in distinguishing patients with NSTEMI from those with UA [Figure 1]. The AUC was 0.576 and did not reach statistical significance (*p* = 0.253), indicating poor discriminatory performance of PAPP-A for differentiating NSTEMI from UA [Table 3].

**Figure 2:**
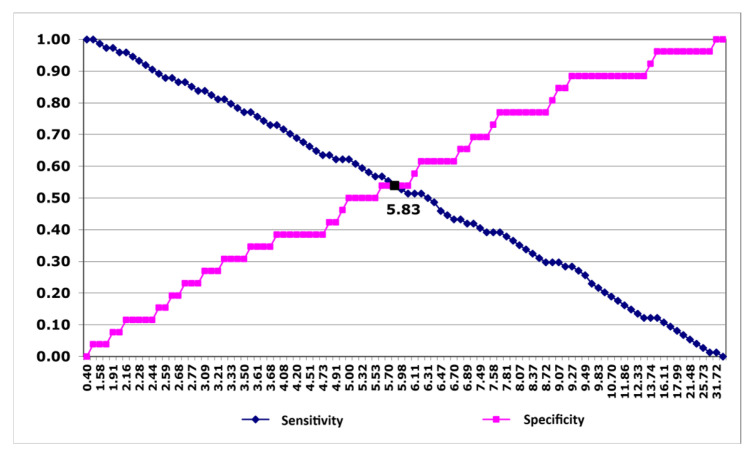
Curves estimating the sensitivity and specificity of PAPP-A protein concentrations for prediction of NSTEMI occurrence.

Further analysis of the above curve also determined the threshold value of PAPP-A protein concentration, allowing for prediction of NSTEMI occurrence. The cut-off point for the PAPP-A protein value turned out to be 5.83 mIU/L [Figure 2]. For this PAPP-A protein concentration, the OR was then calculated. The results are presented in Table 4 (OR and 95% CI for OR are given). Patients with PAPP-A concentration ≥ 5.83 mIU/L had a 1.37-fold higher risk of NSTEMI than those with PAPP-A < 5.83 mIU/L. However, as the 95% CI crosses 1.0, the calculated OR is not statistically significant.

### 3.2. Comparison of Sensitivity, Specificity and Predictive Values of PAPP-A Protein Versus the cTnT for Predicting NSTEMI

For the PAPP-A protein concentration determined during the ROC curve analysis, which allows for the prediction of NSTEMI (5.83 mIU/L) and for the classic marker of myocardial necrosis, i.e., cTnT, the following measures were calculated to determine the usefulness of the diagnostic test: sensitivity, specificity, chance of an FP result, chance of an FN result, PPV, and NPV [Table 5].

The second cTnT test (cTnT-2) provides the best results in predicting myocardial infarction (NSTEMI). The PAPP-A protein with the optimal cut-off point turns out to be an even worse indicator than the first cTnT test. The sensitivity and specificity of the PAPP-A protein test are approximately 54%. The PPV is quite high, which means that in the group of patients with a PAPP-A protein concentration ≥ 5.83 mIU/L, the percentage of patients with myocardial infarction (NSTEMI) was 76.92%.

### 3.3. Assessment of the Associations Between PAPP-A Protein Concentrations and the Occurrence of Adverse Cardiovascular Events

**Figure 3:**
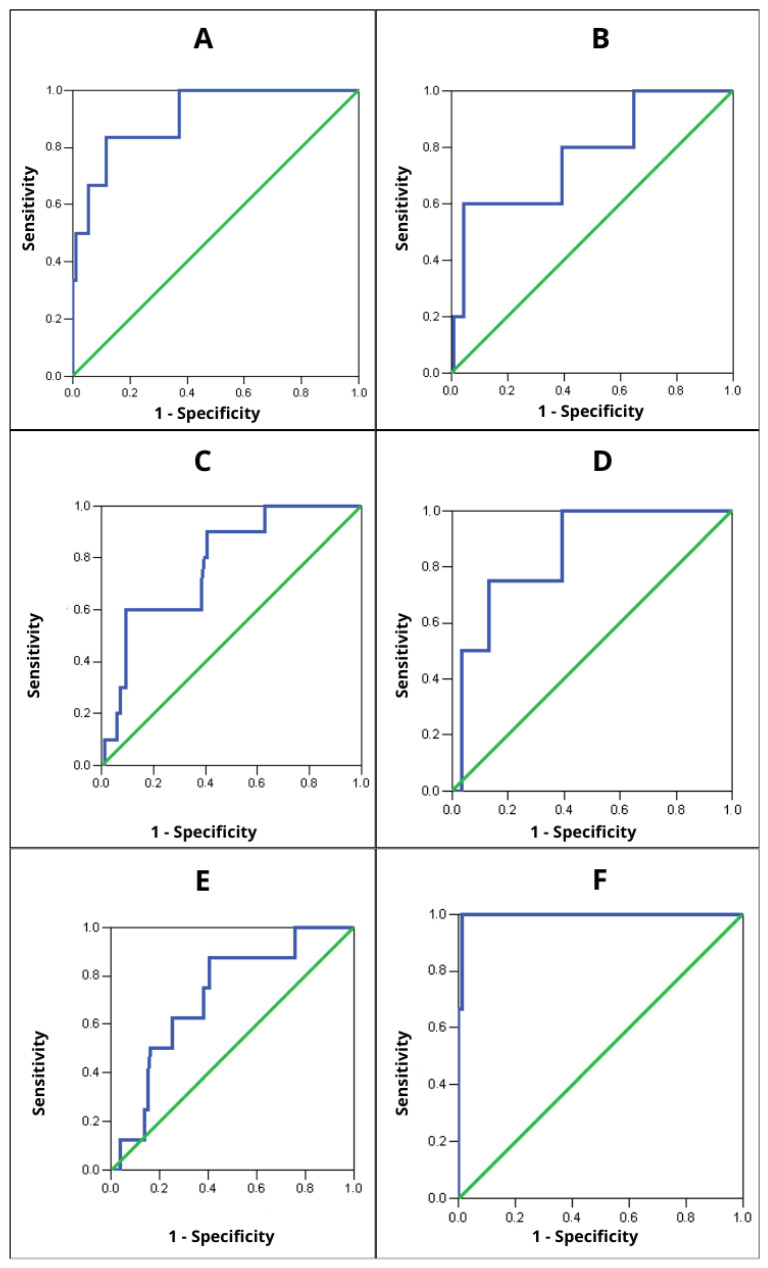
ROC curves for PAPP-A protein concentrations in patients with and without particular adverse events in the specific time points of the observation period. (**A**) Tested variable: PAPP-A protein concentration in differentiating patients in whom a composite endpoint (CE) occurred in the 1st month of observation. (**B**) Tested variable: PAPP-A protein concentration in differentiating patients in whom acute myocardial infarction (AMI) occurred between the 1st and the 3rd month of observation. (**C**) Tested variable: PAPP-A protein concentration in differentiating patients with the need to perform unplanned coronary angiography (UCA) between the 1st and the 3rd month of observation. (**D**) Tested variable: PAPP-A protein concentration in differentiating patients in whom CE occurred between the 3rd and the 6th month of observation. (**E**) Tested variable: PAPP-A protein concentration in differentiating patients with the need to perform UCA between the 3rd and the 6th month of observation. (**F**) Tested variable: PAPP-A protein concentration in differentiating patients in whom CE occurred between the 6th and the 12th month of observation.

Clinical follow-up over 12 months included 97 patients, as three participants from the NSTEMI subgroup died due to ACS during the initial hospitalization. Owing to the relatively small number of adverse cardiovascular events, event data across individual follow-up time points were pooled for both groups. We also constructed a composite endpoint (CE) comprising AMI and CD. ROC curve analyses and AUC statistics for PAPP-A concentration as a discriminator between patients with and without specific adverse events are presented in Figure 3 and Table 6. PAPP-A cut-off values that were significantly associated with the risk of CE or individual adverse events at specific follow-up time points, together with diagnostic performance measures (sensitivity, specificity, PPV, and NPV), are summarized in Table 7.

#### 3.3.1. First Month of the Clinical Follow-Up Period

In the first month of the observation period, CE occurred in three patients (one case of AMI and two cases of CD). In patients with CE in the first month of follow-up, the PAPP-A protein concentration determined in the ACS was significantly higher (*p* < 0.001) than in patients without CE. The analysis shows that this variable differentiates patients at risk of AMI or CD in an excellent way [Table 6]. Further evaluation of the ROC curve revealed a PAPP-A protein concentration cut-off point at 11.44 mIU/L that significantly correlated with the risk of occurrence of CE in the first month of observation. This value was characterized by high sensitivity and specificity. The probability that CE will not occur in subjects with a PAPP-A protein concentration of <11.44 mIU/L is also very high [Table 7]. The predictive value of a positive result of a given diagnostic test for the occurrence of CE was also determined based on the OR. For this cut-off point, the OR was 37.73, which means that the risk of CE was almost 38 times higher than in the case when the PAPP-A protein concentration was <11.44 mIU/L. The calculated confidence interval [95% CI: 8.14–74.83] is very wide, which may result from the relatively small sample size. Therefore, the actual OR of the whole population might differ, although it remains statistically significant.

#### 3.3.2. Interval Between the First and the Third Month of the Clinical Follow-Up Period

In a follow-up assessment conducted between the first and the third month of the observation period, we noted five cases of AMI and six cases of UCA among participants. Statistically significant associations between PAPP-A protein concentration and the risk of AMI (14.59 ± 9.17 mIU/L vs. 6.73 ± 4.92 mIU/L; *p* = 0.042) and the need for UCA (11.64 ± 6.65 mIU/L vs. 6.62 ± 5.08 mIU/L; *p* = 0.004) were observed [Table 6]. In the case of both aforementioned adverse events, the value of AUC indicates that PAPP-A protein differentiates patients in a satisfactory manner. The optimal cut-off points (PAPP-A protein concentrations), determined during further analysis of the ROC curve, for AMI and UCA prediction are presented in Table 7. Particularly high probability of AMI occurrence was found in subjects with PAPP-A protein concentration ≥ 16.34 mIU/L, who presented almost 33-times-higher risk compared to patients with a PAPP-A protein concentration below this value [OR = 32.63; 95% CI: 7.52–141.48]. Participants with a PAPP-A protein concentration ≥ 10.7 mIU/L displayed almost 15-times-higher risk of UCA necessity [OR = 14.63; 95% CI: 4.30–49.69]. Because statistical power was limited by the small number of events, the 95% CIs were relatively wide, limiting the precision of OR estimates in the general population. Nevertheless, as the lower bounds of the 95% CIs exceeded 1.0, these associations remained statistically significant.

#### 3.3.3. Interval Between the Third and the Sixth Month of the Clinical Follow-Up Period

During the period between the third and the sixth month of the clinical follow-up, we recorded five CE events (four patients experienced AMI and one died) and six cases of UCA. Statistically significant associations were observed between PAPP-A concentration and the risk of CE (14.93 ± 7.85 mIU/L vs. 6.80 ± 5.10 mIU/L; *p* = 0.017) as well as the need for UCA (8.86 ± 4.63 mIU/L vs. 6.77 ± 5.25 mIU/L; *p* = 0.044). ROC analysis indicated that admission PAPP-A concentration discriminated patients who required UCA acceptably, while its discriminatory performance was very good for identifying patients at risk of AMI or cardiovascular death [Table 6]. The optimal cut-off values for CE and UCA prediction, along with their sensitivity, specificity, PPV and NPV, are presented in Table 7. Patients with PAPP-A ≥ 10.14 mIU/L had 20-fold higher odds of CE compared with those below this threshold (OR = 20.0; 95% CI: 3.45–115.96), although the estimate was limited by the wide CI. For UCA, PAPP-A ≥ 6.47 mIU/L was associated with more than three-fold higher odds (OR = 3.21; 95% CI: 0.80–12.94); however, because the lower bound of the 95% CI crossed 1.0, this association was not statistically significant.

#### 3.3.4. Interval Between the Sixth and the Twelfth Month of the Clinical Follow-Up Period

During the final 6 months of the clinical follow-up, we noted four cases of CE (three participants experienced AMI and one person died). A relationship was found between PAPP-A protein concentration and the risk of CE (6.40 ± 4.13 mIU/L vs. 24.53 ± 4.23 mIU/L; *p* = 0.004). The analysis clearly shows that the PAPP-A protein concentration determined on admission is a variable that differentiates patients at risk of AMI or CD in a perfect way [Table 6]. The best cut-off point for CE occurrence, determined after looking more closely at the ROC curve, is presented in Table 7 along with its sensitivity, specificity, PPV and NPV.

#### 3.3.5. Association Between PAPP-A Protein Concentration and the Occurrence of Restenosis

**Figure 4:**
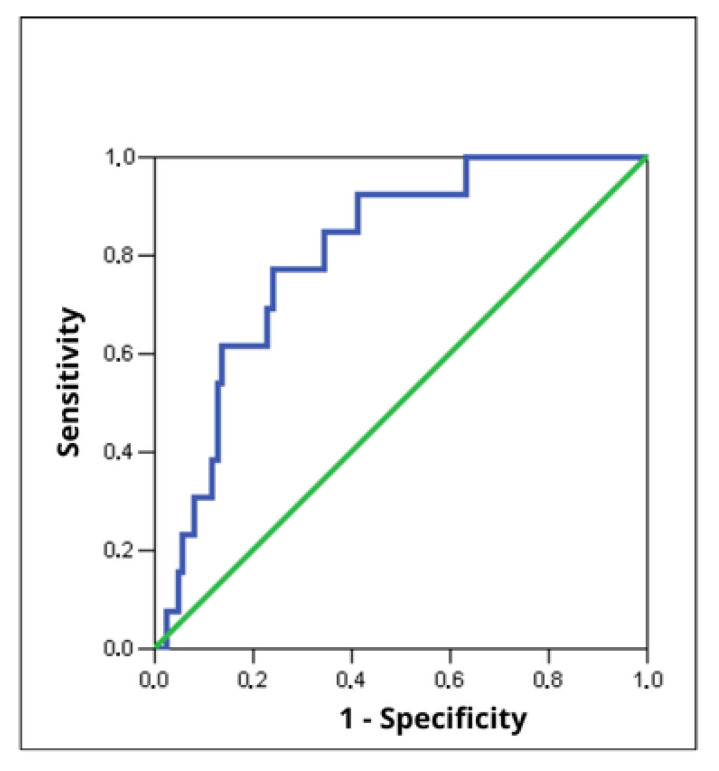
ROC curve—tested variable: PAPP-A protein concentration in differentiating patients who developed restenosis during the 12-month-long clinical follow-up.

During the 12-month-long observation period, we noted 13 cases of restenosis among participants of the study. A statistically significant difference in PAPP-A protein concentration was observed between patients with restenosis and those without restenosis (11.89 ± 6.13 mIU/L vs. 6.92 ± 5.90 mIU/L; *p* = 0.0005) in the 12-month follow-up. Table 8 presents the basic descriptive statistics of the ROC curve [Figure 4], which describe the usefulness of PAPP-A protein concentration in predicting restenosis.

As it results from the above analysis, the PAPP-A protein concentration determined on admission is a variable differentiating patients at risk of restenosis in a very good way. The optimal cut-off point of PAPP-A protein concentration, along with its sensitivity, specificity, PPV and NPV, is presented in Table 9. In patients with a PAPP-A protein concentration ≥8.17 mIU/L, the risk of restenosis was more than 10-fold higher [OR = 10.48; 95% CI: 3.16–34.77], although the accuracy of this estimation is limited by the relatively wide 95% CI.

## 4. Discussion

Every year, over 7 million individuals worldwide experience ACS [6]. In Poland, approximately 100,000 patients were treated for ACS in 2019, and NSTE-ACS accounted for nearly 75% of cases, making it the leading cause of ACS-related hospitalization [39,40]. Moreover, recent observations show a decreasing proportion of STEMI cases in high-income countries, leading to an even more pronounced dominance of NSTE-ACS occurrence [41]. Considering the large number of patients, the variety of symptoms, and the different prognosis in individual groups of patients with NSTE-ACS, early risk stratification, aimed at identifying both patients at high risk of death or adverse cardiovascular events and “low risk” patients, in whom expensive and potentially hazardous invasive strategies offer limited benefit, is crucial. The ECG continues to play a fundamental role in assessing patients with ACS. Biochemical tests have been part of the diagnostic toolkit since the 1950s. The basis for establishing the dominant role of cTn in the diagnosis of patients with ACS was the announcement of a new definition of AMI in 2000 by the European Society of Cardiology (ESC) and the American College of Cardiology (ACC) [42]. Currently, the latest guidelines refer to the Fourth Universal Definition of Myocardial Infarction [38].

In our own studies, elevated cTnT concentration on admission was detectable in 57% of subjects, which also included 77% of patients with a final diagnosis of NSTEMI. Despite its indisputable advantages, cTn concentration determination in the diagnosis of ACS is not free from limitations. Therefore, an ideal marker of necrosis/ischemia, characterized by high sensitivity, specificity, and acceptable cost, is still sought [43]. During the years 2007–2008 in which our study cohort was collected, the diagnosis of ACS was based on cTnT assays with lower analytical sensitivity and precision compared with modern high-sensitivity cardiac troponin (hs-cTn) assays. cTnT assays typically had higher limits of detection and often could not measure troponin concentrations below clinical decision levels, meaning that only more pronounced myocardial necrosis was identified as elevated troponin. The diagnostic threshold used historically for cTnT in many laboratories was aligned with manufacturer-defined cut-offs (in the case of our study it was 0.014 ng/mL), chosen without the rigorous analytical precision criteria later applied to hs-cTn assays. In contrast, hs-cTn assays are designed to have a total coefficient of variation (CV) ≤ 10% at the 99th percentile upper reference limit and to measure troponin above the limit of detection in a large proportion of healthy individuals, enabling earlier and more sensitive detection of myocardial injury and small changes in troponin levels [44,45].

PAPP-A protein is considered to be a promising marker. Researchers observed that in ACS, the PAPP-A marker of plaque instability is significantly elevated [46]. What is more, serum PAPP-A level elevations may occur before or even in the absence of myocardial necrosis [47]. In our own studies, no significant difference in PAPP-A protein concentrations was found between patients with NSTEMI and UA [Table 2]. Similar observations were reported by You et al. (2.42 mIU/L vs. 2.33 mIU/L; *p* > 0.05) and Bayes-Genis et al. (14.9 mIU/L in patients with UA vs. 20.6 mIU/L in patients with AMI; *p* = 0.5) [8,48]. In contrast, a statistically significant difference in PAPP-A protein concentrations between patients (30.3 mIU/L in STEMI, 21.13 mIU/L in NSTEMI and 19.73 mIU/L in UA, respectively, *p* = 0.045) was demonstrated by Hájek et al. [49]. Their report stands in line with the observations of Mehrpooya et al. In their study, PAPP-A protein concentrations were significantly lower in subjects with UA compared to those with STEMI or NSTEMI [50]. An analysis conducted by Schaub et al. also revealed that individuals with AMI have higher concentrations of PAPP-A than those with other diagnoses, including UA [4.6 mIU/L vs. 4.0 mIU/L, *p* < 0.001]. However, no significant differences were observed in PAPP-A levels between STEMI and NSTEMI [51]. These discrepancies may be partly explained by methodological differences. Initially, PAPP-A protein concentration was determined using tests commonly utilized in gynaecology for screening for Down syndrome [52]. Eventually, it was noticed that the concentration of PAPP-A protein in pregnant women is many times higher than in men and non-pregnant women [9,51], and it can even reach values of 3655 mIU/L [53]. Therefore, it became necessary to create tests capable of detecting low concentrations of PAPP-A protein (so-called “ultra sensitive” tests). The commonly available tests may contain antibodies reacting with both the PAPP-A protein and proMBP, or antibodies adequate only for the PAPP-A protein molecule. Thus, the former detect the PAPP-A protein in the form of a tetramer (2 PAPP-A protein molecules bound to 2 proMBP molecules). A direct consequence of the aforementioned properties is therefore the detection of PAPP-A protein originating from cells of the female reproductive system, kidneys, large intestine and bone marrow [54,55,56] and the possibility of failure to detect the ACS-specific PAPP-A homodimer [14,57]. Assays using antibodies directed only against PAPP-A are increasingly common; however, structural alterations of PAPP-A in the absence of proMBP may still hinder detection of ACS-associated forms [14,57]. This obstacle can be overcome with diagnostic measures specially manufactured to detect PAPP-A protein molecules specific for ACS (taking into account particular epitopes), although they are not commercially available [13,57,58]. Therefore, it is not surprising that we observed the aforementioned discrepancy in PAPP-A protein concentrations. Moreover, the lack of statistically significant differences between NSTEMI and UA subgroups might result from their common pathophysiological background. PAPP-A is released from activated macrophages and vascular smooth muscle cells at sites of disrupted or inflamed atherosclerotic plaques, a process that occurs in both UA and NSTEMI. Because plaque rupture or erosion with superimposed thrombosis represents a shared upstream pathophysiological mechanism in these syndromes, circulating PAPP-A levels may rise irrespective of whether downstream myocardial necrosis is sufficient to produce detectable troponin release. This commonality likely explains why PAPP-A lacks diagnostic discrimination between UA and NSTEMI yet retains promising prognostic value by reflecting the underlying burden of biologically active, rupture-prone plaques that predispose to recurrent ischemic events [8,59].

In our study we found that subjects who displayed PAPP-A protein concentrations ≥ 5.83 mIU/L were potentially at greater risk of NSTEMI. However, the exploratory diagnostic test utilizing this cut-off point of PAPP-A protein concentration was less useful for diagnosing NSTEMI than the first cTnT test. The sensitivity, specificity and PPV of the PAPP-A protein test were lower than corresponding measures for the above cTnT test. For comparison, Bayes-Genis et al. indicated that a PAPP-A protein concentration >10 mIU/L allowed for the identification of patients with ACS, achieving a sensitivity close to 90% and a specificity exceeding 80% [8]. A Czech study found increased PAPP-A in heparin-naive ACS patients with high PPV (95.7%) and lower NPV (47.7%). In NSTE-ACS, the AUC did not differ from that for troponin I (TnI) [60]. On the other hand, a study by Schaub et al. considers PAPP-A to be of little value in the diagnosis of ACS, with an AUC of 0.61, which was inferior to troponin [51]. Slightly higher PAPP-A protein concentration values, allowing for identification of patients with ACS with the highest sensitivity and specificity, were reported by Hájek et al. [49]. For individual types of ACS, these values ranged between 10.65 and 14.75 mIU/L (with the AUC ranging from 0.613 to 0.919). The absence of hs-cTn testing in our cohort may have influenced patient classification. Some patients with mild myocardial injury—detectable by hs-cTn but not by older assays—could have been classified as troponin-negative. This misclassification potentially alters the relative diagnostic performance observed for PAPP-A in relation to troponin. hs-cTn assays have been shown to outperform conventional cTnT in predicting major adverse cardiac events and improve early rule-out strategies, particularly when serial measurements and 99th percentile thresholds are applied [61,62]. In the era of hs-cTn, the incremental value of PAPP-A may not be as pronounced for the initial diagnosis of ACS, given the high sensitivity and earlier detection afforded by modern troponin assays. However, PAPP-A might still contribute additional prognostic information by identifying patients with vulnerable plaques and heightened risk of future events beyond myocardial injury alone [63].

The time from the onset of symptoms to the moment of blood collection for the determination of PAPP-A protein concentration varied in individual studies. In our own studies, blood was collected after the patients were admitted to the hospital (8 ± 3.6 h from the onset of pain in patients with NSTEMI and 9.5 ± 2.7 h among patients with UA). This was pretty much the same as the time it took to collect blood in the study by Bayes-Genis et al., but different from that in the Czech–German studies (in STEMI—4 ± 3.3 h; in NSTEMI—9.1 ± 2.1 h; in UA—15.5 ± 9.5 h) [8,49]. The data on the kinetics of PAPP-A protein concentrations are rather modest, although it appears that in the case of STEMI they normalize rapidly [64,65,66], while in NSTE-ACS the elevated concentrations remain at a similar level for 24–36 h [66,67,68]. The noted discrepancy in measured PAPP-A protein levels may potentially be affected by other variables, such as heparin, often used in ACS treatment. The capacity of the PAPP-A protein to bind heparin has been recognized for an extended period, and the precise binding site was delineated in 2004 by Weyer et al. [69,70]. In vitro, heparin and PAPP-A protein are known to compete for binding on the cell surface [71]. Subsequent research has unequivocally shown an elevation in PAPP-A protein levels due to the effects of both unfractionated heparin and low-molecular-weight heparins [33,72,73]. The precise mechanism of this phenomenon remains unidentified. One hypothesis posits that the fast elevation in PAPP-A protein concentration upon heparin administration, regardless of the type, results from the release of PAPP-A protein from the endothelial cell surface in arteries with very high blood flow. In our research, one of the exclusion criteria was the administration of unfractionated or low-molecular-weight heparin treatment. This exclusion criterion was not applied in Bayes-Genis et al. and the investigations of Hájek et al. [8,49]. The most up-to-date ACS treatment regimens recommend routine application of heparin in treatment of both STE-ACS and NSTE-ACS, especially in patients undergoing angiography and PCI [74]. This leads to heparins being frequently administered in the prehospital setting or immediately after admission to hospital. Consequently, PAPP-A measurements obtained after initiation of anticoagulant therapy may predominantly reflect the pharmacological effect of heparin rather than true plaque instability or disease activity [33,75]. In the light of the referenced findings, this significantly limits the diagnostic utility of PAPP-A in routine clinical practice, particularly when the exact timing of blood sampling relative to heparin administration cannot be reliably determined. These observations also highlight the need for careful standardization of sampling time and adjustment for heparin exposure in statistical analyses of future studies evaluating the prognostic role of PAPP-A [33,75].

Troponins are not only critical in diagnosing individuals with ACS, but they also serve as an exceptional prognostic tool. Extensive data supporting the efficacy of their determination in risk assessment for patients with NSTE-ACS have established cTn as the foundation for risk classification in this cohort [6,76,77,78]. In view of a number of reports on the significance of PAPP-A protein as a prognostic factor in patients with ACS [27,46,79,80,81,82], our own studies assessed its association with adverse events in a 12-month follow-up. Given the small number of adverse cardiovascular events observed, they were considered in the entire NSTE-ACS group. A CE including AMI and CD was also constructed.

In the first month of clinical follow-up, a relationship was observed between the concentration of PAPP-A protein determined in ACS and the risk of the occurrence of CE (20.59 mIU/L vs. 6.73 mIU/L; *p* = 0.0008). The cut-off point of the concentration of PAPP-A protein estimated during further analysis was associated with a nearly 38-fold higher risk of CE. It was also characterized by very high sensitivity and specificity, while the probability that at the concentration of PAPP-A protein < 11.44 mIU/L CE would not occur was almost 99%. A similar cut-off point, with the same endpoint, was determined by Heeschen et al., whereby in the case of PAPP-A protein concentration ≥ 12.6 mIU/L the risk of CD or AMI was only just over twice as high [59]. Such a significant difference may be due primarily to the different numbers and characteristics of the study groups. The cohort examined by Henshen et al. was significantly larger than our own (323 vs. 100 ACS subjects). Our relatively small sample size, combined with a low incidence of adverse events, may have contributed to an overestimation of the effect size and the resulting high OR observed in our analysis [59]. In Bonaca et al.’s study, PAPP-A levels exceeding 6.0 μIU/mL were associated with increased rates of CD or AMI at 30 days (7.4% vs. 3.7%) and at 1 year (14.9% vs. 9.7%). At 30 days, PAPP-A was also associated with increased incidences of AMI and CD [63]. In the same observation period, Laterza et al. also noticed a relationship between PAPP-A protein concentration and the risk of a complex endpoint including CD, AMI and the need for revascularization. However, the threshold concentration value determined was much lower (0.22 mIU/L) than in our assessment and was also characterized by lower sensitivity and specificity (66.7% and 51.1%, respectively). Such a large discrepancy in PAPP-A protein concentration should be associated with the use of a different test (American company DSL) for the determination [83].

In our own studies, a statistically significant relationship was observed in the 3-month follow-up between the PAPP-A protein concentration and the risk of AMI or the need for UCA. Among patients with a PAPP-A protein concentration ≥ 16.34 mIU/L, the risk of AMI was almost 33 times higher, and the risk of UCA was almost 15 times higher in the case of a PAPP-A protein concentration ≥ 10.7 mIU/L. Similarly, a relationship between PAPP-A protein level and the risk of adverse events (AMI or death) during a 3-month follow-up was found by Iversen et al., who evaluated 415 patients with low-risk NSTE-ACS (no ECG changes and negative cTn test result). Danish investigators found that in the case of PAPP-A protein level > 12.4 mIU/L (evaluated in most patients from two or three blood samples), the risk of AMI or death was 3.7 times higher [68]. Similarly to the case of 1-month observation, this high discrepancy between OR calculated in our analysis and results acquired by other investigators may be dictated by disparities in sample size. The relatively low number of participants in our assessment likely inflated our effect size, leading to the high OR.

During the next observation period (up to 6 months), our own examination found a significant relationship between PAPP-A protein concentration and the risk of CE or the need for UCA (8.86 mIU/L vs. 6.77 mIU/L; *p* = 0.044). After exceeding the optimal cut-off point, the risk of CE was 20 times higher. This value was characterized by quite high sensitivity and specificity. What is particularly interesting is that at a PAPP-A protein concentration < 10.14 mIU/L, the probability of not developing CE was almost 99%. In the case of a PAPP-A protein concentration < 6.47 mIU/L, the probability of not having to perform UCA was 99%, and after exceeding this value, the risk increased more than 3-fold. Heeschen et al. and Lund et al. also assessed the risk of adverse events during a 6-month follow-up. One study found that PAPP-A protein levels above 12.6 mIU/L increased the risk of developing a CNS or AMI by more than 2.5 times. The latter indicated that the concentration of PAPP-A protein > 2.9 mIU/L was associated with a more than 4.5-fold increase in the risk of the endpoint including CD, AMI, and the need for revascularization [59,84]. However, the methodology for determining the PAPP-A protein concentration used in the studies by Lund et al. (immunofluorometric method) and Heeschen et al. (immunoenzymatic method—using a reagent manufactured by Roche) differed from that used in our own studies [59,84].

In our own studies, during the observation of patients in the period of 6–12 months after ACS, a relationship between the concentration of PAPP-A protein and the risk of the occurrence of CE was found again, with the optimal cut-off point for CE prediction set at ≥19.43 mIU/L. Similarly, analysing the factors influencing the prognosis of patients with STEMI during a 12-month follow-up period, Lund et al. found that a PAPP-A protein concentration above 10 mIU/L was associated with an increased risk of CD and AMI, while Iversen et al. found a 2.4-fold increase in the risk of AMI or death among patients at low risk of NSTE-ACS in the case of PAPP-A protein concentration > 12.4 mIU/L [64,68]. Both studies used averaged PAPP-A values from multiple measurements, whereas our analysis was based on a single admission sample measured by sandwich ELISA [64,68].

When analysing adverse events in the observation of patients after ACS, it is impossible not to take restenosis into account. Restenosis is one of the main limitations of PCI—a widely used and effective treatment strategy in ACS [85,86,87,88,89,90]. Angiographically significant restenosis is defined as ≥50% narrowing of the vessel lumen at the site of the previous PCI procedure [91]. According to estimates, up to 50% of patients may experience restenosis [92,93,94]. This complication may manifest as recurrence or exacerbation of exertional angina and/or ischemic changes in the resting or exercise ECG [95,96]. This usually occurs between the first and the sixth month after PCI, with some authors claiming that the peak of incidence falls in the period between the third and the sixth month after PCI [97]. This aligns with our findings, as clinical indications for repeat coronary angiography occurred most frequently in the 3–6-month interval of clinical follow-up. Restenosis was angiographically confirmed in all cases with clinical suspicion. Of 13 restenosis cases observed overall, 10 occurred within the first 6 months. Reports on the clinical course of restenosis are contradictory. Many authors state that in most patients it manifests as ACS [98,99]. In our own studies, ACS was a clinical manifestation of restenosis in 85% of cases [in 54.55%, AMI; in the remainder, UA]. These data are comparable with the observations of Bainey et al. (ACS was a clinical manifestation of restenosis in 70.7%, of which NSTE-ACS constituted 52.2% and STEMI 18.5%), but different from the data gathered by the Cleveland Clinic registry (ACS was a clinical manifestation in 35.9% of restenoses, of which AMI constituted 9.5% and UA 26.4%) [100,101]. In our own studies, a statistically significant difference in PAPP-A protein concentration was found in patients with and without restenosis. PAPP-A protein concentration determined on admission turned out to be a variable differentiating patients at a very high risk of restenosis. The risk of restenosis was more than 10-fold higher when the PAPP-A protein concentration was ≥8.17 mIU/L. To our knowledge, comparative data on this association remain limited in the available literature.

## 5. Limitations

This study has several limitations related to the period in which the data were collected. First, troponin was measured using non-high-sensitivity assays, which are less sensitive than modern hs-cTn assays and may have failed to detect minor myocardial injury. As a result, some patients with small or early myocardial infarctions may have been misclassified as troponin-negative, potentially influencing diagnostic comparisons and the observed performance of PAPP-A. Because hs-cTn testing was not available, the findings may not be directly generalizable to populations managed under current diagnostic algorithms [61]. Furthermore, management of NSTEMI during the study period differed substantially from current practice, with less routine use of early invasive strategies, contemporary antiplatelet agents, and guideline-directed secondary prevention. Advances in PCI techniques, including improved stent technology and procedural strategies, may have altered clinical outcomes and risk profiles, thereby limiting the direct applicability of our prognostic findings to present-day ACS cohorts [74,102].

The type of test we used to determine the PAPP-A protein represents a significant limitation of our own research. We used a commercially available test with high sensitivity, enabling the detection of low PAPP-A protein concentrations. However, it contains antibodies that react with both the PAPP-A protein molecule and proMBP. The choice of the test was dictated primarily by promising literature data on the usefulness of “ultra sensitive” tests in assessing PAPP-A protein concentrations in patients with ACS [83,103]. Furthermore, as previously mentioned, tests dedicated to detecting the specific epitopes present in the PAPP-A protein homodimer are not commercially available.

The study included a relatively small number of patients, specifically only 26 in the UA group, which led to a decrease in the power of the diagnostic tests. Univariate analyses failed to confirm the influence of specific individual factors on adverse events. Multivariate analysis was not performed. Additionally, the number of adverse cardiovascular events during 12-month follow-up decreased. However, we jointly analysed these events in both subgroups and created composite endpoints. The small sample size and relatively low number of adverse cardiovascular events in analysed groups might have overstated the effect size in OR calculations, leading to wide 95% CIs and their high upper range values. Moreover, the lower the sample size, the more susceptible it is to random data fluctuations, which in the case of our research might have contributed to disturbances in the analysis of optimal cut-off points for adverse cardiovascular event prediction and limit the precision of their sensitivity and specificity estimation.

The incidence of adverse cardiovascular events discussed in this paper has multifactorial origin, including compliance to long-term, post-MI treatment, dynamics of lipid profile, or previously implemented PCI strategy [104,105,106]. Therefore, an analysis of PAPP-A’s potential as a single biomarker of atherosclerotic plaque instability and risk of future thrombotic events or restenosis should be viewed as exploratory, demanding validation in combination with other exponents of cardiovascular risk and in larger, multi-centre cohorts.

## 6. Conclusions

Elevated PAPP-A concentrations in patients with NSTE-ACS appeared, in this small single-centre cohort, to have only modest diagnostic value, performing less well than troponin T (threshold ≥ 5.83 mIU/L); however, the limited sample size restricts the reliability of this comparison. Within the same cohort, higher PAPP-A levels were indicatively associated with increased risks of several adverse outcomes, although these estimates should be interpreted with caution due to limited statistical power. Concentrations ≥ 8.17 mIU/L showed a potential 10-fold increase in 12-month restenosis risk, while values ≥ 16.34 mIU/L suggested an approximately thirty-three-fold elevation in 3-month myocardial infarction risk. Similarly, thresholds of ≥11.44 mIU/L (1 month), ≥10.14 mIU/L (6 months), and ≥19.43 mIU/L (12 months) were suggestively associated with a more than 20-fold increase in the composite endpoint of myocardial infarction or cardiovascular death. PAPP-A levels ≥ 10.7 mIU/L showed a tentative association with worsening coronary artery disease and requiring repeat angiography within 3 months. These findings should be viewed as exploratory and hypothesis-generating rather than definitive, pending validation in larger, multi-centre studies.

## Figures and Tables

**Table 1:** Demographic, clinical and biochemical characteristics in patients with NSTEMI and UA.

Parameter		Patients with NSTEMI (*n* = 74)	Patients with UA (*n* = 26)	*p*
Age (years)		63.39 ± 10.52	66.50 ± 10.27	0.196 **
Male sex		48 [64.86%]	20 [76.92%]	0.591 *
Dyslipidaemia		74 [100.0%]	26 [100.0%]	1.0 *
Hypertension		69 [93.20%]	23 [88.46%]	0.439 *
Smoking		61 [82.43%]	21 [80.77%]	0.849 *
Obesity		29 [39.19%]	9 [34.62%]	0.679 *
Overweight		32 [43.24%]	10 [38.46%]	0.672 *
T2DM		30 [40.54%]	11 [42.31%]	0.874 *
Impaired fasting blood glucose		20 [27.03%]	8 [30.77%]	0.715 *
Familial occurrence of atherosclerotic diseases		14 [18.92%]	5 [19.23%]	0.972 *
Marginal irregularities in the coronary arteries		5 [6.76%]	6 [23.07%]	0.022 *
Critical stenoses in the coronary arteries		69 [93.24%]	20 [76.91%]	0.022 *
Critical stenoses in	1 coronary artery	29 [39.19%]	6 [23.07%]	0.138 *
	2 coronary arteries	15 [20.27%]	7 [26.92%]	0.481 *
	≥3 coronary arteries	25 [33.78%]	7 [26.92%]	0.519 *
EF value [%]		56.85 ± 10.15	57.73 ± 13.81	0.374 *
Number of patients with	EF ≥ 55%	51 [68.9%]	19 [73.1%]	0.374 *
	40% ≤ EF < 55%	19 [25.7%]	4 [15.4%]	0.374 *
	EF < 40%	4 [5.4%]	3 [11.5%]	0.374 *
Glycemia [mg/dL]		141.97 ± 78.06	125.19 ± 77.23	0.187 **
TC [mg/dL]		201.92 ± 45.17	188.12 ± 43.37	0.179 ***
LDL [mg/dL]		123.97 ± 41.89	112.38 ± 38.32	0.218 ***
HDL [mg/dL]		50.54 ± 20.70	47.85 ± 19.76	0.207 **
TG [mg/dL]		135.47 ± 75.06	149.62 ± 76.74	0.301 **
Creatinine [mg/dL]		0.89 ± 0.24	0.90 ± 0.22	0.396 **
Uric acid [mg/dL]		6.44 ± 1.09	6.48 ± 1.86	0.934 ***
CRP [mg/L]		7.54 ± 7.56	7.40 ± 10.15	0.359 **
Fibrinogen [g/L]		4.69 ± 2.11	5.13 ± 2.40	0.376 **
Leukocytes [10^3^/mm^3^]		8.89 ± 2.83	8.03 ± 2.48	0.132 **
Neutrophils [%]		61.79 ± 10.50	61.37 ± 8.37	0.857 **

CRP—C reactive protein; EF—ejection fraction; HDL—high-density lipoproteins; LDL—low-density lipoproteins; NSTEMI—non-ST-elevation myocardial infarction; TC—total cholesterol; TG—triglycerides; T2DM—type 2 diabetes mellitus; UA—unstable angina. Obesity was defined as body mass index (BMI) value ≥ 30. Overweight was defined as 30 > BMI value ≥ 25. Smoking was defined as smoking at least 5 cigarettes per day for a year, without stopping earlier than 6 months before the date of hospital admission. The data are expressed as the number of observations with a given variable variant (*n*) and its corresponding percentage (%) or mean value ± standard deviation (SD). The bolded results indicate statistically significant differences. * *p*-value assessed using the chi-square test. ** *p*-value assessed using the Mann–Whitney U test. *** *p*-value assessed using Student’s T-test.

**Table 2:** Comparison of PAPP-A protein levels in patients with NSTEMI and UA.

PAPP-A Protein Concentration [mIU/L]	Patients with NSTEMI (*n* = 74)	Patients with UA (*n* = 26)	*p* *
Mean	7.93	6.52	0.253
Median	6.31	5.26	
Min	1.44	0.60	
Max	35.84	27.60	
Q25	3.62	3.04	
Q75	9.58	7.60	
SD	6.35	5.45	

NSTEMI—non-ST-elevation myocardial infarction; PAPP-A—pregnancy-associated plasma protein A; UA—unstable angina. The data are expressed as mean, median, minimum, maximum, IQR (Quartile 1; Quartile 3) and standard deviation (SD). * *p*-value assessed using the Mann–Whitney U test.

**Table 3:** Basic descriptive statistics of the ROC curve describing the usefulness of PAPP-A concentration in differentiating patients with NSTEMI and UA.

AUC	SE	*p* *	95% CI	
			Lower Limit	Upper Limit
0.576	0.065	0.253	0.449	0.703

The data are expressed as area under the curve (AUC), standard error (SE) mean and confidence level (CI) with lower and upper limit. * *p*-value assessed using the chi-square test.

**Table 4:** OR and 95% confidence interval for NSTEMI occurrence among patients with PAPP-A concentration ≥ 5.83 mIU/L.

Diagnostic Test	OR	−95% CI	+95% CI
PAPP-A ≥ 5.83 mIU/L	1.37	0.56	3.36

PAPP-A—pregnancy-associated plasma protein A. The data are expressed as odds ratio (OR) and confidence level (Cl).

**Table 5:** Comparison of sensitivity, specificity and predictive values of cTnT and PAPP-A protein in predicting NSTEMI.

Diagnostic Test	Sensitivity	Specificity	PPV	NPV	Chance of Results	
					False Positive	False Negative
cTnT-1 positive	58.11%	100.00%	100.00%	45.61%	0.00%	41.89%
cTnT-2 positive	100.00%	100.00%	100.00%	100.00%	0.00%	0.00%
PAPP-A ≥ 5.83 mIU/L	54.05%	53.85%	76.92%	29.17%	46.15%	45.95%

cTnT—cardiac troponin T; NPV—negative predictive value; PAPP-A—pregnancy-associated plasma protein A; PPV—positive predictive value. The data are expressed as the percentage (%) of observations.

**Table 6:** Basic descriptive statistics of the ROC curves for PAPP-A protein concentrations in prediction of particular adverse events at specific time points during the clinical follow-up period.

Tested Variable	Adverse Event	Cases of Adverse Events in Subgroups		Number of Participants Still in the Follow-Up	AUC	SE	*p* *	95% CI	
		NSTEMI	UA					Lower Limit	Upper Limit
PAPP-A protein concentration in the first month of the observation period	CE	2	1	97	0.908	0.057	**0.001**	0.797	1.019
PAPP-A protein concentration between the first and the third month of the observation period	AMI	5	0	95	0.771	0.116	**0.042**	0.544	0.999
	UCA	5	1	95	0.777	0.070	**0.004**	0.641	0.914
PAPP-A protein concentration between the third and the sixth month of the observation period	CE	5	0	95	0.853	0.078	**0.017**	0.700	1.006
	UCA	5	1	95	0.716	0.083	**0.044**	0.554	0.879
PAPP-A protein concentration between the sixth and the twelfth month of the observation period	CE	3	1	94	0.996	0.006	**0.004**	0.986	1.007

AMI—acute myocardial infarction; CE—composite endpoint; NSTEMI—non-ST-elevation myocardial infarction; PAPP-A—pregnancy-associated plasma protein A; UA—unstable angina; UCA—unplanned coronary angiography. The data are expressed as area under the curve (AUC), standard error (SE) mean and confidence level (CI) with lower and upper limit. * *p*-value assessed using the chi-square test. The bolded results indicate statistically significant differences.

**Table 7:** The PAPP-A protein concentration cut-off points that significantly correlated with the risk of particular adverse events at specific time points during the clinical follow-up period and their basic measures determining the usefulness of a given diagnostic test.

Time of Adverse Event Occurrence	Type of Adverse Event	Diagnostic Test	Sensitivity	Specificity	PPV	NPV
The first month of the observation period	CE	PAPP-A protein concentration ≥ 11.44 mIU/L.	83.33%	88.30%	31.25%	98.81%
Between the first and the third month of the observation period	AMI	PAPP-A protein concentration ≥ 16.34 mIU/L	60.00%	95.60%	42.86%	97.75%
	Necessity of UCA	PAPP-A protein concentration ≥ 10.7 mIU/L	60.00%	90.70%	42.86%	95.12%
Between the third and the sixth month of the observation period	CE	PAPP-A protein concentration ≥ 10.14 mIU/L	75.00%	86.96%	20.00%	98.77%
	Necessity of UCA	PAPP-A protein concentration ≥ 6.47 mIU/L	66.67%	61.63%	15.38%	94.64%
Between the sixth and the twelfth month of the observation period	CE	PAPP-A protein concentration ≥ 19.43 mIU/L	100%	98.91%	75.00%	100%

AMI—acute myocardial infarction; CE—composite endpoint; NPV—negative predictive value; PAPP-A—pregnancy-associated plasma protein A; PPV—positive predictive value; UCA—unplanned coronary angiography.

**Table 8:** Basic descriptive statistics of the ROC curve assessing PAPP-A protein concentration in predicting restenosis during 12 months of the clinical follow-up.

Adverse Event	AUC	SE	*p* *	95% CI	
				Lower Limit	Upper Limit
Restenosis occurring within 12 months of clinical follow-up	0.802	0.055	**0.0005**	0.694	0.910

The data are expressed as area under the curve (AUC), standard error (SE) mean and confidence level (CI) with lower and upper limit. * *p*-value assessed using the chi-square test. The bolded results indicate statistically significant differences.

**Table 9:** Sensitivity, specificity, PPV, and NPV for PAPP-A concentration ≥ 8.17 mIU/L in predicting restenosis during 12 months of clinical follow-up.

Diagnostic Test	Sensitivity	Specificity	PPV	NPV
PAPP-A protein concentration ≥ 8.17 mIU/L	76.92%	75.86%	32.25%	96.65%

NPV—negative predictive value; PAPP-A—pregnancy-associated plasma protein A; PPV—positive predictive value.

## Data Availability

The datasets used and/or analysed during the current study are available from the corresponding author on reasonable request.
